# Maximum effect with minimum impact: A new selective control strategy for the Browsing ant *Lepisiota frauenfeldi* (Formicidae: Formicinae) minimize the impact on non-target species

**DOI:** 10.1371/journal.pone.0337230

**Published:** 2025-12-03

**Authors:** Masashi Yoshimura, Mayuko Suwabe, Kazuki Tsuji, Yosuke Baba, Hiroshi Kakazu, Tatsuya Miyagi, Masako Ogasawara, Jumpei Uematsu, Evan P. Economo, Koji Ono

**Affiliations:** 1 Environmental Science and Informatics Section, Okinawa Institute of Science and Technology Graduate University, Onna, Kunigami, Okinawa, Japan; 2 Ryukyu Nature Positive, Independent Consultant, Uruma, Okinawa, Japan; 3 University of the Ryukyus, Nishihara, Nakagami, Okinawa, Japan; 4 SC Environmental Science Co., Ltd., Takarazuka, Hyogo, Japan; 5 Naha City Hall, Naha, Okinawa, Japan; 6 The United Graduate School of Agricultural Sciences, Kagoshima University, Kagoshima City, Kagoshima, Japan; 7 Biodiversity and Biocomplexity Unit, Okinawa Institute of Science and Technology Graduate University, Onna, Kunigami, Okinawa, Japan; 8 Department of Entomology, University of Maryland, College Park, Maryland, United States of America; 9 Okinawa Amami Nature Conservation Office, Ministry of the Environment, Naha, Okinawa, Japan; Universidade Federal de Uberlandia - Campus Umuarama, BRAZIL

## Abstract

Early-stage control of invasive ants provides an opportunity to suppress establishment while conserving existing biodiversity. We developed and tested a selective control strategy targeting the Browsing ant *Lepisiota frauenfeldi*, an emerging omnivorous, polygynous, and polydomous invader, during its initial establishment in an urban area of Okinawa Island, Japan. Because the local ant community was still present when the invasion was detected, the trial aimed at reducing the competitive advantage of the target species while preserving biotic resistance from resident species. We hypothesized that weakening *L*. *frauenfeldi* through selective chemical treatment would allow existing species to occupy its vacant niches, thereby accelerating eradication. Species-specific baits were formulated using a growth regulator with protein- and carbohydrate-based substrates. Baits were placed near nest entrances identified through monthly surveys over an area of approximately 1.6 hectares. Ant community dynamics were monitored for 44 months using hand-collection and sticky-trap surveys. The invasive ant showed a strong negative response to treatment, with an estimated 92% reduction in occurrence probability, ultimately disappearing from the treated area. Several non-target ant species increased in frequency, consistent with expectation of the niche reoccupation, while most other species remained stable, indicating minimal impact on the broader community. Compared to conventional methods, this approach used drastically less chemical while achieving high efficacy. Implementation through cross-organizational collaboration enabled parallel treatment in adjacent restricted areas and contributed to the program’s success. These results highlight the practical and ecological value of nest-targeted, community-collaborative selective baiting as a strategy for managing omnivorous invasive ants during early establishment.

## Introduction

It is known that the extent of biotic resistance from existing ants’ communities to invasive ants varies regionally [1]. When invasive alien ants excel in terms of food source discovery, recruitment, and duration of activity, native species are likely to be displaced [1], resulting in local ant communities changing to simple ones dominated by invasive alien species. In such cases, when the problem becomes apparent, the invasive alien species is already dominant in these habitats, and it is prioritized to control invasive species rather than preserving remaining other ant species [2]. Even if other species are not considered, it will be primarily the invasive alien ants being controlled that will be affected by the baits applied at many such sites because of the monopolization of food sources by the invaders [3]. On the other hand, areal and indiscriminate chemical application in the early stages of invasion, when local ant communities have not been eradicated, may instead undermine resistance by existing local ant species [3]. Furthermore, there has been shown that disturbance to native ant communities by chemical application and soil tillage promotes the establishment of invasive alien ant colonies [4–6]. As such, it has been argued that native ant communities need to be restored to prepare for the re-invasion of invasive alien species after the control process [7]. In an example of the Red-Imported Fire Ant, *Solenopsis invicta* Buren, 1972, one of the strongest invasive alien ants, the threshold for population establishment bottlenecks is found in the early to small stages of colony development [6]. Thus, we should focus on selective chemical treatment to reduce the colony size of the target species to below the threshold while preserving the existing ant communities. It may accelerate the eradication of the invasive alien ant’s colonies by existing biological resistance. Interspecific competition between native and invasive alien ant species is known to occur [2], and native ants ecologically similar to the invasive alien species that compete for the same niche are expected to be most susceptible [1]. However, on the other hand, those “susceptible groups” in the existing ant community could be the strongest preventers for invasive alien species once the power balance between the two sides is reversed. Maintaining local bioresistance is also expected to be effective in preventing the re-establishment of invasive alien species in areas threatened by re-invasion from neighboring areas. Furthermore, a strategy of selective chemical application to target ant species for control has the potential to significantly reduce the absolute amount of chemicals used in an area, a benefit that helps minimize the environmental impact. Alder and Silverman [2] state that effective management of a particular species without interference from non-target ants may be possible by considering food preference, colony boundaries, and diel activity patterns. Such attempts at selective chemical application to target species for control have been successfully implemented in *Brachyponera chinensis* (Emery, 1895) control, which is known as a dietary specialist termite eater, and in the dominant environment of *Linepithema humile* (Mayr, 1868) [8–10]. In the above attempts, live fipronil-treated termites were used as baits, and small amounts of the chemical were highly effective in controlling the ants. However, there has been no success in dealing with omnivory, a common feature of many invasive alien ants [1], in situations where the forces of multiple species are competing in the early stages of an invasion.

Here, we attempted to selectively control *Lepisiota frauenfeldi* (Mayr, 1855) under the conditions of early establishment with the presence of the local ant community consisting of many species. This technology of selective control could have a wide range of applications for many other invasive ant species that share common features: omnivorous diet, occupying multiple nests (polydomy), and having multiple queens in a colony (polygynous). However, several issues need to be resolved with existing baiting methods to achieve selective control under these conditions. In the early stages of establishment, when the ant community in the area is still competitive to the invader, the usual baiting method is likely to carry away a certain amount of baits by non-target species and weaken the existing community [3]. It would also be difficult to maximize selective bait delivery to target species based solely on differences in prey preference or hunting behavior, as used in control a dietary specialist (as in Buczkowski [8]). On the other hand, the success of weakening the target species by selective chemical application may reverse the competitive strength between the target invasive ant species and other ant species in the existing community, thereby accelerating the control of the target. This reversal is expected to appear as a decline in the target species and the occupation of the vacant ecological niches by existing species. It is simultaneously expected that preventing the re-establishment of the target invasive species from surrounding areas through maintaining biological resistance through the existing ant community. We solved these issues by optimizing bait application methods and bait recipes based on the foraging behavior and food preferences of the target species, *L*. *frauenfeldi*. The control effectiveness on the target species and the reduction of its impact on non-target species were demonstrated by the dynamics on the occurrence frequency of each species. The frequency data is taken by sticky trapping and hand-collecting surveys, for the latter was conducted following the modified Time Unit Sampling protocol [11]. We also attempted to address social issues to control implementation, which can be an obstacle to the control, especially in densely populated areas, by establishing a cooperative system among the related agencies. We hypothesize that selective chemical control targeting the invasive ant *L*. *frauenfeldi*, when optimized based on its foraging behavior and food preferences, will (1) reduce the target species’ population below the stable maintenance thresholds, (2) minimize impacts on non-target resident ant species, and (3) facilitate the recovery of existing competing ant communities, thereby enhancing biotic resistance contributing to the acceleration of the its eradication and prevention of re-invasion.

## Materials and methods

### Research site

**Fig 1:**
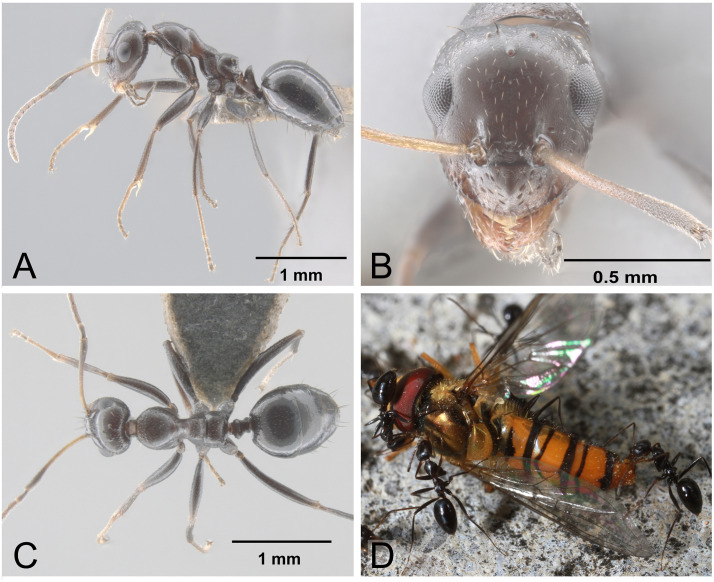
*Lepisiota frauenfeldi* established on Okinawa-jima. (A) body in lateral view; (B) head in full-face view; (C) body in dorsal view; (D) foraging workers.

**Fig 2:**
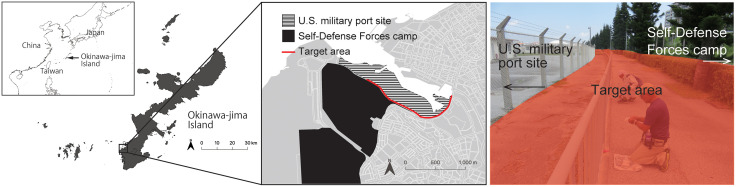
Research site. The target area was an approximately 1.8 km stretch on national roads 331 and 332 connecting Naha International Airport and the center of Naha City. The entire north side of the target area borders Naha Port under the control of **U.**S. Army Garrison Okinawa, and a portion of the south side borders a Self-Defense Forces camp. This map was created by the authors using the following open data sources: Natural Earth (public domain); National land use data from MLIT, Japan; Land use data from Okinawa Prefectural Government.

The control area of the present study is located in Okinawa-jima, Japan. The island is located in the subtropical region, and the climate is classified as Cfa under the Köppen system. According to meteorological data from Naha station, the average temperature from January 2020 to December 2024 was 23.9 ± 4.3°C (mean ± SD, n = 60 months). During 2020–2024, the monthly average of low temperatures in the area was 21.7 ± 4.4°C, and that of high temperatures was 26.5 ± 4.3°C (mean ± SD, n = 60 months). The annual average precipitation was 2664.7 ± 345.9 mm (mean ± SD, n = five years). Okinawa-jima is the largest island in the “Ryukyu Archipelago” (as defined in Toyama [12], Fig 7) and is known for high endemism, making it an important island for biodiversity conservation [13]. While the northern forest area was designated as a World Natural Heritage Site in 2021, leading to strengthened conservation, an urban area with a population of over one million occupies the south-central region. Logistics hubs centered on the international ports and airports in the region are at risk of invasion by alien species, as has been observed at other logistics hubs [14]. The establishment of *L*. *frauenfeldi* in Okinawa was confirmed for the first time on the national road in Naha City by a monitoring survey of alien ant species conducted by the Ministry of the Environment in February 2020 [15] and was later discovered at Naha Shinko Wharf in a subsequent investigation (Fig 1). The target area of *L. frauenfeldi* treatment in this study was over an approximately 1.8 km stretch of the busy national roads 331 and 332 between Naha International Airport and the center of Naha City (Fig 2). The total target area was estimated to be approximately 16,100 m^2^ (1.61 ha). Naha City is the largest city and urban center on Okinawa-jima, with a population of over 300,000. The entire north side of the target area borders Naha Port under the control of U.S. Army Garrison Okinawa (c.a. 55.9 ha), and a portion of the south side borders a Self-Defense Forces camp. Initial discovery of *L*. *frauenfeldi* was in a planted area on the north sidewalk between the roadway and the base. *L*. *frauenfeldi* were then found nested in roadway curb cracks and at high density in drainage pipes on the slope of the road throughout the target area. A number of individuals were also frequently observed coming and going from maintenance holes.

### Target species

The target species of the present study was *L*. *frauenfeldi*, which were established at the treatment area. *L*. *frauenfeldi* is considered to have a negative impact on the diversity of the local ant and other insects. This ant species is listed as invasive by the Food and Agriculture Organization [16] and as the target of a control program in Australia. Similar to some serious invasive ant species, *L. frauenfeldi* exhibits high aggressiveness to other species and gains dominance over the competitors through the population size and density because of its polydomy and polygyny. Being omnivorous, the food menu overlaps with those for many existing ant species. It is observed that it recruits nestmates to monopolize and defend the resource once a food source is discovered. In 2020, this ant species was designated as an invasive alien species under the Invasive Alien Species Act of Japan and as a priority control species on the Okinawa Prefecture Invasive Alien Species List in 2021 [17]. *L*. *frauenfeldi* is thought to be native to southern Europe; however, its spread around the world is already underway, with invasions found in Réunion, Australia, Guam, Taiwan, and Japan [18,19]. In Japan, establishment was first confirmed in Aichi Prefecture in 2017 [19], followed by Tokyo, Osaka, and Fukuoka Prefectures in the same year [15]. Further findings in Kagoshima, Hyogo, and Kanagawa Prefectures in 2019 and Okinawa and Shizuoka Prefectures in 2020 [15] indicate a remarkable expansion of the invasion area.

We should mention that the target species of this study was identified as *Lepisiota frauenfeldi*, but as with the other Japanese populations, this is tentative for practical reasons. The taxonomic treatment of this species relative closely related groups seems to be still insufficient [20] to clarify the strict species boundaries. Even though all reported populations found in Japan were keyed out to the single species in the existing identification keys, the morphological variations were observed among established populations in Japan, as between Okinawa (Fig 1) and Osaka (Figs 1, 2 in Ueda and Murakami [15]). All ant species (Table 1), including *L*. *frauenfeldi* collected in this study, were identified based on morphological comparison with the ant collection at the Okinawa Institute of Science and Technology Graduate University (OIST; from the OKEON Churamori Project insect collection), Japan, in addition to using the Ant Database Group 2008 [21], and other identification keys [22–24].

**Table 1:** Ant species and the number of collected plots (frequency of occurrence) within the surveyed plots.

		hand-collecting																						sticky trap																					
		2020						2021												2022				2020						2021												2022			
Subfamily	Species	Jul	Aug	Sep	Oct	Nov	Dec	Jan	Feb	Mar	Apr	May	Jun	Jul	Aug	Sep	Oct	Nov	Dec	Jan	Feb	Mar	Apr	Jul	Aug	Sep	Oct	Nov	Dec	Jan	Feb	Mar	Apr	May	Jun	Jul	Aug	Sep	Oct	Nov	Dec	Jan	Feb	Mar	Apr
Dolicoderinae	*Tapinoma melanocephalum*	31	17	14	15	15	15	17	13	16	12	16	16	27	16	17	18	19	19	18	18	19	16	18	12	7	10	8	3	5	3	5	3	9	8	16	7	10	–	7	8	4	11	2	5
Formicinae	*Anoplolepis gracilipes*	0	0	0	0	0	1	0	0	0	1	2	2	5	2	2	2	2	0	2	2	2	2	1	0	0	1	0	0	0	0	0	2	2	0	4	2	2	–	2	2	1	1	2	2
	*Camponotus bishamon*	4	1	2	2	1	0	0	1	1	1	2	3	4	3	2	2	1	0	1	2	1	1	0	0	0	0	0	0	0	0	1	1	0	0	0	0	0	–	0	0	0	0	0	0
	*Colobopsis shohki*	0	0	0	0	0	0	0	0	0	0	0	0	0	0	0	0	0	2	0	0	0	1	0	0	0	0	0	0	0	0	0	1	0	0	0	0	0	–	0	0	0	0	0	0
	*Lepisiota frauenfeldi*	24	16	8	2	2	2	1	2	0	7	5	8	3	0	0	0	2	1	2	0	0	0	16	7	2	1	0	0	0	0	0	2	6	3	3	0	0	–	0	0	0	0	0	0
	*Nylanderia amia*	15	14	7	12	4	12	11	7	12	9	10	15	16	11	18	17	15	14	12	5	16	15	11	4	3	6	4	2	0	0	0	4	5	3	12	1	10	–	5	4	1	4	3	5
	*Nylanderia* OK03	0	0	0	2	9	0	0	0	0	5	1	1	0	1	0	0	0	0	1	0	0	1	0	0	0	0	0	0	0	0	0	0	0	0	0	0	0	–	0	0	0	0	0	0
	*Paratrechina longicornis*	26	14	12	16	17	16	12	13	10	13	13	16	16	13	17	16	14	14	13	13	15	15	26	13	9	13	8	3	2	3	5	6	10	9	12	11	6	–	7	8	3	2	4	7
	*Plagiolepis alluaudi*	1	2	1	3	2	1	1	1	1	1	2	2	2	2	3	2	3	2	2	2	3	3	0	0	0	0	0	0	0	0	0	1	1	1	2	0	1	–	0	2	1	1	0	1
Myrmicinae	*Cardiocondyla kagutsuchi*	3	1	0	5	4	3	4	1	1	10	5	10	4	4	7	11	6	8	5	5	5	7	1	1	1	0	0	0	0	0	0	1	2	2	11	2	2	–	0	0	0	0	0	0
	*Cardiocondyla minutior*	8	7	9	8	11	3	9	2	5	6	9	7	11	6	8	14	11	7	5	4	4	8	1	1	1	0	1	0	0	1	0	2	1	3	5	2	2	–	0	2	0	0	1	0
	*Cardiocondyla obsucurior*	0	0	0	0	0	0	0	0	0	0	0	1	0	0	1	0	0	0	0	0	0	0	0	0	0	0	0	0	0	0	0	0	0	0	0	0	0	–	0	0	0	0	0	0
	*Crematogaster vagula*	2	1	2	0	0	0	0	0	0	1	1	1	2	2	3	1	1	0	0	0	2	3	0	0	0	0	0	0	0	0	0	0	0	0	0	0	0	–	0	0	0	0	0	0
	*Monomorium chinense*	32	19	17	15	17	6	15	9	12	20	19	19	33	19	20	19	16	18	12	14	19	20	13	7	10	7	4	2	3	0	1	3	11	9	16	5	5	–	4	3	0	0	0	4
	*Monomorium floricola*	6	5	8	6	8	2	3	1	0	5	5	5	5	6	7	8	9	9	1	4	2	8	4	1	1	0	1	0	0	0	0	1	2	1	1	1	0	–	3	3	0	0	1	2
	*Syllophopsis sechellensis*	1	0	0	0	0	1	0	0	0	1	0	0	0	0	0	2	0	0	0	0	0	0	0	0	0	0	0	0	0	0	0	0	0	0	0	0	0	–	0	0	0	0	0	0
	*Trichomyrmex destractor*	3	0	0	0	0	0	0	0	0	0	0	1	2	1	0	0	0	0	0	0	0	0	4	1	1	0	0	0	0	0	0	1	1	0	0	0	0	–	0	0	0	0	0	0
	*Pheidole indica*	2	2	2	2	2	1	3	1	2	2	1	3	0	3	1	2	2	2	3	2	3	2	2	2	2	2	2	1	1	0	2	3	2	2	3	0	0	–	2	1	0	1	0	2
	*Pheidole megacephala*	11	5	7	7	7	7	7	8	7	7	6	3	5	0	3	1	6	5	6	6	4	5	5	3	3	6	4	5	7	1	3	4	4	0	2	3	3	–	0	5	1	2	0	2
	*Pheidole parva*	16	10	12	6	11	4	5	1	3	6	6	10	17	13	14	13	14	11	1	1	10	10	16	4	8	6	2	3	2	2	1	3	8	9	8	7	6	–	5	1	1	0	6	2
	*Tetramorium bicarinatum*	7	4	4	3	5	4	2	3	2	3	2	2	4	2	7	4	7	2	3	1	4	1	8	5	7	7	2	2	4	1	1	3	2	5	7	5	10	–	1	6	2	1	3	2
	*Tetramorium lanuginosum*	2	3	2	0	3	0	0	0	1	1	2	3	2	1	3	2	0	0	0	0	0	0	0	0	0	0	0	0	0	0	0	1	0	1	1	1	0	–	0	0	0	0	0	2
	*Tetramorium simillimum*	1	2	0	2	1	2	0	1	1	1	2	3	0	0	1	2	3	0	1	0	0	0	0	0	0	0	0	0	0	0	0	1	0	1	0	0	0	–	0	0	0	0	0	0
	*Tetramorium smithi*	0	0	0	0	0	0	0	0	0	0	0	1	0	0	0	0	0	0	0	0	0	0	2	0	0	0	0	0	0	0	1	1	2	0	0	1	1	–	0	0	0	0	0	0
Ponerinae	*Brachyponera chinensis*	0	0	0	0	0	0	0	0	0	0	0	0	0	0	0	0	0	0	0	0	0	0	0	0	0	0	0	0	0	0	0	1	0	1	2	0	1	–	0	0	0	0	0	0

The numbers in July are those in 36 plots, while those in the other months are those in 20 plots. The lack of data on sticky traps in October 2021 was the cause of the traps being stolen before collection.

### Bait agent recipe

We had to develop our own baits since we found that the bait attractiveness of *L*. *frauenfeldi* was unstable. *L*. *frauenfeldi* were not attracted to the fipronil ant control baits available in Japan, at least from the winter to the early summer: the former is when the population was discovered first in Okinawa, and the latter is when chemical control of the established population started. We chose to use baiting agents mixed with an Insect Growth Regulator (hereafter, IGR) that would have a milder treatment effect but would be less disturbing their nest environment, and we further increased the efficiency of ingestion by separating the substrate into a carbohydrate and protein (Tsuji in prep.). Pyriproxyfen was selected as the IGR, which has a proven track record in controls against *L*. *frauenfeldi* [19]. A solution with 0.1% pyriproxyfen was provided by SC Environmental Science Co., Ltd. Since *L*. *frauenfeldi* were not interested in any of the conventional ant-treating baits with pyriproxyfen distributed in Japan, we selected attractants based on the ease of handling and the results of preliminary experiments on their food preferences. Out of the two, one was mealworms as the protein-biased attractant, and the other was white sugar water as the carbohydrate-biased attractant. Mealworms were first frozen for a short time before being injected with 0.1 mL of the pyriproxyfen solution using a syringe. Six individuals were scissored and placed in each application point, i.e., 0.0006 mg pyriproxyfen/ point. In preliminary experiments using up to a maximum concentration of 50% (w/w), the higher the sugar concentration, the more *L*. *frauenfeldi* were attracted and, conversely, the higher the pyriproxyfen concentration, the more the ants were repelled. Therefore, we made a 50% (w/w) sugar and 0.025% (w/w) pyriproxyfen solution by adding the equivalent weight of white sugar to a pyriproxyfen solution adjusted to 0.05% (w/w) with water. We then soaked pieces of 25 mm square cotton in 4 mL aliquots of the pyriproxyfen solution and placed four pieces of the cotton at each point, i.e., 0.004 mg pyriproxyfen/ point.

### Bait application method

We located the entrances to the *L. frauenfeldi* nests and placed baits near them to effectively deliver bait to the target species, while minimizing the impact on non-target ant species. We conducted monthly visual surveys of the approximately 1.8 km establishment range to identify suspicious individuals in the field. Once the individuals were confirmed as being the target ants, we sought to locate their nest entrances in order to place the baits. When the entrances could not be found, baits were placed in areas with higher densities of *L*. *frauenfeldi*. We also placed baits in areas with fewer individuals once the density of ants had dropped off to one to several per point and had become difficult to trace to the nest. Both protein and carbohydrate baits were applied up to twice every month from August 2020 to February 2022 on dispensing papers fixed to the road surface with curing tape depending on the visual survey result. The twice bait applications in a month were conducted from September 2020 to September 2021 and from November 2021 to February 2022 between the monthly visual surveys, and the same points as the first application were used for the second one. The number of bait application points varied depending on the number of nest entrances located and the number of points where the target ants had been confirmed, and no bait was applied in October 2021 and after February 2022 because we could not find any individual of the target ant by the visual survey (Table 2). Visits to the baits by *L*. *frauenfeldi* were pictured 30 minutes after application and counted to evaluate the attraction of the installed baits.

**Table 2:** Detailed information about bait installation and the amount of Pyriproxyfen in it.

	Bait installation (1st / 2nd)																					
	2020						2021												2022			
	Jul	Aug	Sep	Oct	Nov	Dec	Jan	Feb	Mar	Apr	May	Jun	Jul	Aug	Sep	Oct	Nov	Dec	Jan	Feb	Mar	Apr
Sugar bait	NA / NA	41 / NA	32 / 32	13 / 13	9 / 9	2 / 2	2 / 2	4 / 3	1 / 1	7 / 7	11 / 11	20 / 20	6 / 6	4 / 4	1 / 1	0 / 0	4 / 4	5 / 5	9 / 9	2 / 2	0 / 0	0 / 0
(points)																						
Pyriproxyfen	0.0	164.0	256.0	104.0	72.0	16.0	16.0	28.0	8.0	56.0	88.0	160.0	48.0	32.0	8.0	0.0	32.0	40.0	72.0	16.0	0.0	0.0
(µg)																						
Mealworm bait	NA / NA	35 / NA	32 / 32	12 / 12	8 / 8	1 / 1	1 / 1	3 / 4	0 / 0	6 / 6	10 / 10	20 / 20	6 / 6	4 / 4	1 / 1	0 / 0	4 / 4	5 / 5	9 / 9	2 / 2	0 / 0	0 / 0
(points)																						
Pyriproxyfen	0.0	21.0	38.4	14.4	9.6	1.2	1.2	4.2	0.0	7.2	12.0	24.0	7.2	4.8	1.2	0.0	4.8	6.0	10.8	2.4	0.0	0.0
(µg)																						
Pyriproxyfen	0.0000	0.1850	0.2944	0.1184	0.0816	0.0172	0.0172	0.0322	0.0080	0.0632	0.1000	0.1840	0.0552	0.0368	0.0092	0.0000	0.0368	0.0460	0.0828	0.0184	0.0000	0.0000
Total (mg)																						

Twice installation a month were started from September 2020. In the early stage, a sugar bait and mealworm bait were placed alternately but were set those side to side from June 2021.

### Measuring Effectiveness

To evaluate the effectiveness of the chemical treatment in controlling *L. frauenfeldi* and its impact on other ant species in the area, the approximately 1.8 km long with 9 m width established area was equally divided into 36 plots of about 50 m long each, and the monthly surveys were conducted the plot-based. All 36 plots were surveyed in July 2020–2023, and 20 plots were selected and surveyed in the other months. The selected 20 plots with the highest nesting densities of the target species were chosen based on the result in July 2020. Detection using baits is the most common method of surveying ants; however, preliminary tests showed that it is difficult to attract *L*. *frauenfeldi* consistently regardless of the type of bait used, so this method was not employed. Instead, we used sticky traps, with one sticky trap set in each survey plot every month and collected after 72 h or more [25]. In addition, 10-min hand-collection surveys were conducted in each plot to obtain a more comprehensive view of the fine dynamics of *L*. *frauenfeldi* and the other ant species. The hand-collection survey was a modified version of the 15-min time-unit sampling (TUS) survey of ants [11], which is frequently conducted in the prefecture. During the survey, the investigators tried to maximize the number of species collected within the given time limit. The collected samples were fixed in 99% ethanol on site. All material collected in the sticky traps and hand collections were identified in the laboratory. The hand-collection samples are stored at the OIST as part of the OKEON Churamori Project Arthropods Collection.

Observations in the research area were conducted from July 2020 to February 2024. Out of the 44 months, monthly observations were conducted from its start to August 2023, then November 2023, and February 2024 to confirm the eradication of *L*. *frauenfeldi* in the control area. Out of the observation period, “before-control” was defined as surveys in July and August 2020, and “post-control” was defined as surveys after this period. Data from the start date to April 2022, two months after the last bait application, were used to evaluate the dynamics of *L*. *frauenfeldi* and to assess the impacts on other species caused by the IGR treatments. Ant survey data is recorded as presence (1) and absence (0) for each species in each survey unit that is one trap for the sticky trap survey or one collection event for a ten-minutes hand-collection survey.

Statistical analyses of the survey data were performed using generalized linear mixed models (GLMM) and generalized linear models (GLMs) packages of R (version 4.4.2). The GLMM with a binomial distribution and a logit link function was applied to analyze the effects of the IGR treatment, collection method, and standardized temperature on the presence or absence of *L*. *frauenfeldi* and other ant species in the target area. Treatment, method, and standardized temperature were included as fixed effects, and survey plots were included as random intercepts to account for spatial non-independence. Multiple models were constructed to verify the principal effects, treatment and temperature interactions, and treatment and method interactions. Additionally, separate models were estimated for each collection method to evaluate method-specific responses. Model estimation was performed using the glmer function in the lme4 package, with the optimization algorithm set to “bobyqa” or “Nelder Mead” to ensure convergence. The effects of temperature and bait type on the attracted abundance of *L*. *frauenfeldi* were evaluated using the GLMs with Negative binomial, which showed the smallest value in the Akaike information criterion (AIC). Generalized linear mixed models (GLMMs) with a negative binomial distribution using the glmmTMB package were made to investigate the effects of temperature and bait type on ant abundance. The models included standardized temperature, bait type (mealworm or sugar), and their interaction as fixed effects. Setting sites were included as a random effect to account for spatial non-independence. Separate models for each bait type using the same negative binomial GLMM framework were also established to further assess bait-specific responses to temperature.

## Results

**Fig 3:**
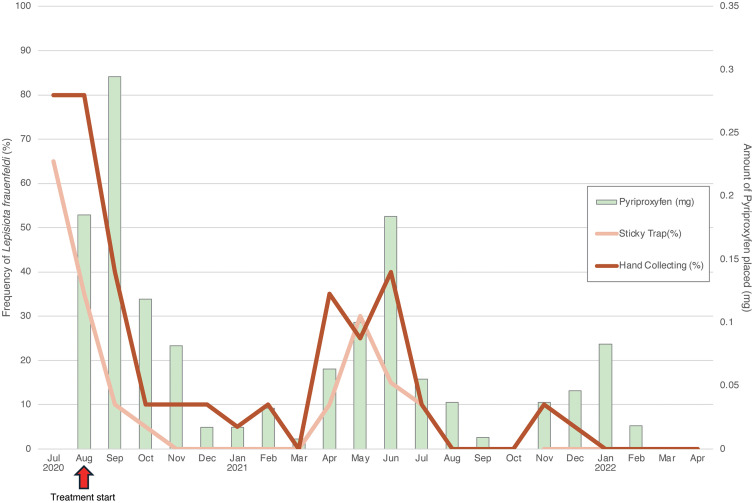
Monthly frequency of occurrence of *Lepisiota frauenfeldi* in the 20 plots of the control area, and the amount of Pyriproxyfen applied. Both hand collections and sticky traps showed a decrease in the frequency of appearance of the target ants due to the application of the Pyriproxyfen baits. In most months, the detection power of the hand-collection method exceeded that of the sticky trap method.

The present study successfully reduced the population of target ant species *L*. *frauenfeldi* (Fig 3) with minimal impacts on the other ant species, and with a small amount of the IGR application. From August 2020 to February 2022, we applied sugar baits at a total of 304 points and mealworm baits at 284 points. The total amount of pyriproxyfen mixed with the baits and applied in the target area during this period was approximately 1.39 mg (0.86 mg/ha), with zero to twice placements every month (Fig 3). The amount of pyriproxyfen treatments per month varied depending on the number of nests or individuals found by the field survey, with the highest amount of 0.29 mg occurring in second month and 0.00 mg in October 2021 and March 2022 (Fig 3).

**Fig 4:**
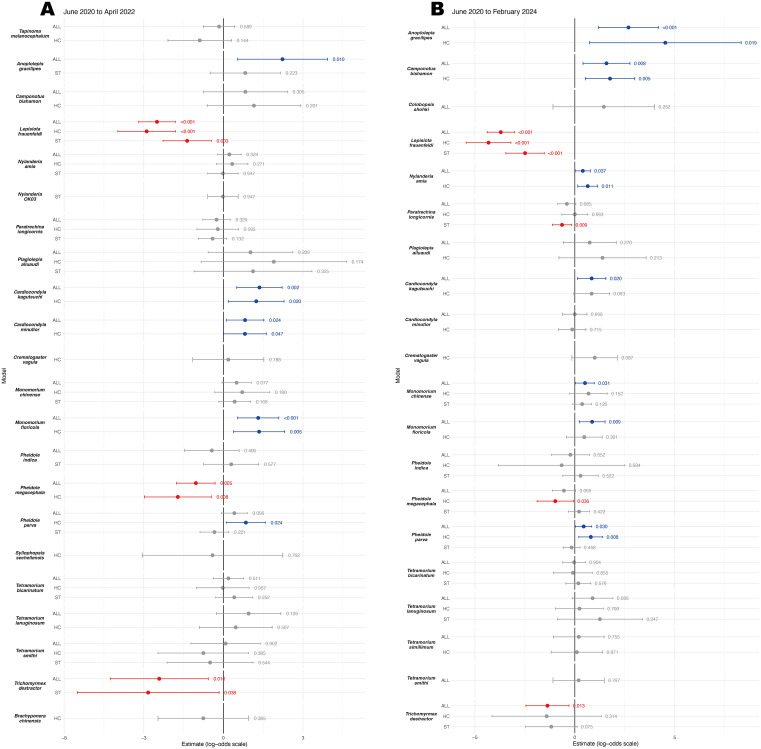
Forest plot of Pyriproxyfen treatment effects for each ant species recorded in the control area including (A) up to two months after the last application (to April 2022), and (B) all survey period (to February 2024). Model estimates for all samples (All), hand-collection (HC), and sticky trap (ST) are shown with 95% confidence intervals on the log-odds scale. Data points with 95% CI upper bounds >6 were omitted from the plot in a; all omitted estimates were not statistically significant.

In the study period from July 2020 to March 2022, three subfamilies, 15 genera, and 24 species of ants, including *L*. *frauenfeldi*, were recorded by the hand-collection surveys, while four subfamilies, 14 genera, and 21 species were recorded by the sticky traps (Table 1). A total of 25 ant species were analyzed to evaluate the effects of IGR treatment (Before vs. After), collection method between Hand-Collection (HC) and Sticky Trap (ST), and standardized temperature using generalized linear mixed models (GLMMs) and at least one model was successfully converged for 22 species (Fig 4). Significant reductions in the occurrence probability after the IGR treatment were detected for three species, *L*. *frauenfeldi*, *Pheidole megacephala*, and *Trichomyrmex destractor*; while significant increases were observed for five species, *Anoplolepis gracilipes*, *Cardiocondyla kagutsuchi*, *C*. *minutior*, *Monomorium floricola*, and *Pheidole parva* (Fig 4). A particularly strong negative response was exhibited after treatment on the target species, *L*. *frauenfeldi*. In the overall dataset, the GLMMs indicated a significant decrease (Estimate = -2.50±0.35 SE, p < 0.001, Odds ratio (OR) = 0.082, 95% CI = -3.191 to -1.810), corresponding to an approximately 92% reduction in the odds of occurrence, and then the species disappeared in the control area since February 2022. Hand-collection (HC) and sticky-trap (ST) surveys showed similar decrease trends (HC: Estimate = -2.89±0.56 SE, p < 0.001, OR = 0.056, 95% CI = -3.980 to -1.802; ST: Estimate = -1.36±0.46 SE, p = 0.003, OR = 0.255, 95% CI = -2.275 to -0.455). In contrast, strong and significant increases after the IGR treatment were indicated in overall dataset of *M*. *floricola* (All: Estimate = 1.31±0.40 SE, p < 0.001, OR = 3.711, 95% CI = 0.535 to 2.088), corresponding to an approximately 3.7 times higher in the odds of occurrence. Hand-collecting (HC) survey showed similar trends (Estimate = 1.35±0.49 SE, p = 0.006, OR = 3.839, 95% CI = 0.381 to 2.310). In addition to the strong reactions, moderate decrease were demonstrated in two species, *P*. *megacephala* (All: Estimate = -1.04±0.37 SE, p = 0.005, OR = 0.355, 95% CI = -1.762 to -0.312; HC: Estimate = -1.72±0.64 SE, p = 0.007, OR = 0.180, 95% CI = -2.975 to -0.458) and *T*. *destructor* (All: Estimate = -2.42±0.94 SE, p = 0.010, OR = 0.089, 95% CI = -4.262 to -0.571; ST: Estimate = -2.83±1.36 SE, p = 0.038, OR = 0.059, 95% CI = -5.507 to -0.162), approximately 65% and 91% reduction in overall dataset, respectively. Although *T*. *destructor* showed an apparent decrease of more than 90% in odds, this result was accompanied by wide uncertainty and modest statistical support. Moderate increases were detected in four species, *A*. *gracilipes* (All: Estimate = 2.23±0.87 SE, p = 0.010, OR = 9.262, 95% CI = 0.529 to 3.923), *C*. *kagutsuchi* (All: Estimate = 1.36±0.44 SE, p = 0.002, OR = 3.895, 95% CI = 0.507 to 2.212; HC: Estimate = 1.24±0.53 SE, p = 0.021, OR = 3.446, 95% CI = 0.191 to 2.284), *C*. *minutior* (All: Estimate = 0.82±0.36 SE, p = 0.024, OR = 2.262, 95% CI = 0.108-1.525; HC: Estimate = 0.81±0.41 SE, p = 0.047, OR = 2.253, 95% CI = 0.010 to 1.615), and *P*. *parva* (HC: Estimate = 0.85±0.38 SE, p = 0.024, OR = 2.332, 95% CI = 0.110 to 1.583). Although these ORs correspond to approximately 9.3 times, 3.9 times, 2.3 times, and 2.3 times higher odds of occurrence, the results are accompanied by wide uncertainty and modest statistical support. Neither positive nor negative effects were detected for the other species.

**Fig 5:**
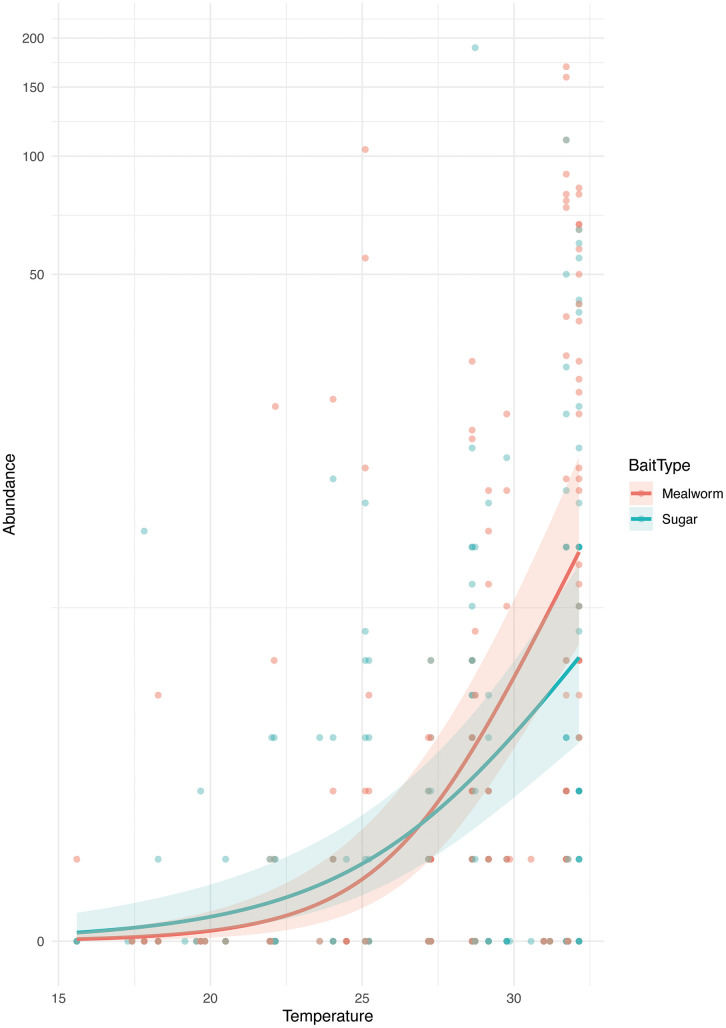
Predicted number of foraging *Lepisiota frauenfeldi* workers in relation to temperature for two bait types: sugar (blue) and mealworm (red). Lines represent model predictions from negative binomial GLMMs fitted separately for each bait type, and shaded areas indicate 95% confidence intervals. Although the predicted abundance increased with temperature for both bait types, the preference patterns appeared to shift around 27°C, where the predicted values for sugar and mealworm baits crossed.

A negative binomial generalized linear mixed model (nbGLMM) was selected based on the AIC value (AIC = 2112.0) to examine the effects of temperature and bait type on the number of visiting foragers of *L*. *frauenfeldi*. The model showed that temperature had a significant positive effect on forager abundance (Estimate = 0.36 ± 0.05 SE, p < 0.001, OR = 1.44). The effect of bait type was marginally significant (Anova: χ² = 3.92, df = 1, p = 0.048). Although the interaction between temperature and bait type was not statistically significant (p = 0.165), the estimated curves for sugar and mealworm baits crossed around 27°C (Fig 5).

## Discussion

In the present study, we succeeded in selective control of *L. frauenfeldi* while it is in the early establishment stage in the presence of many ant species. In countering the omnivorous *L*. *frauenfeldi*, which had not become a sole dominant species, selective control was achievable by the complex of several strategies: improvement in bait formulation based on our ecological knowledge, using low concentrations of IGR, and the selective bait placement close to *L*. *frauenfeldi* nest entrances. We also demonstrated the effectiveness of the control and its impact on non-target species, as shown by differences of the probability of occurrence of each species using hand-collection survey modified the Time Unit Sampling method and sticky trap survey. Although it was not statistically significant, estimated number of visiting foragers of *L*. *frauenfeldi* related with temperature on sugar and mealworm was reversed around 27°C (Fig 5). It may be the reason why the phenomenon of the prey preference of this ant changed depending on the season. Combining the two types of baits may mitigate the reduction in effectiveness of the chemical due to seasonal changes in preference. It took about two months to decrease *L*. *frauenfeldi* in each plot below the detection limit in the study area after start of the IGR treatment. In this trial, we used only pyriproxyfen intending to continuously inhibit adult production in *L*. *frauenfeldi* colonies and reduce their competitiveness with other ant species. The colony size is expected to reduce along with the normal death of component individuals if the pyriproxyfen inhibits only adult production and does not directly kill individuals. On the other hand, the rate of decline in the occurrence probability of *L*. *frauenfeldi* was faster than our expectation. Sakamoto and Goka [26] reported that pyriproxyfen treatment caused not only the termination of new adult production but the mortality of existing adults in *Tetramorium tsushimae* Emery, 1925 colonies. In artificial *T*. *tsushimae* colonies consisting of 100 individuals treated with pyriproxyfen, more than 95% of the worker ants were dead in about 60 days [26]. The drastic decrease in the occurrence probability of *L*. *frauenfeldi* observed about two months after the start of pyriproxyfen treatment suggests that pyriproxyfen killed adults the same as in the case of *T*. *tsushimae*. Zanola et al. [27] reported that populations of *Linepithema humile* detect boric acid in bait and abandon it, suggesting that the apparent reduction in the number of individuals detected by bait may not necessarily reflect a decline in the population size. Meanwhile, regarding the disappearance of *L*. *frauenfeldi* populations within the control area in this study, despite the suggested potential effects of pyriproxyfen on adults, the reductions were detected using both sticky trap and hand-collection methods, and no escaped colony of target species to surrounding areas was confirmed. Further data accumulation is required to answer whether this population reduction is due to the characteristics of pyriproxyfen or the successful maximization of initial uptake [27].

**Fig 6:**
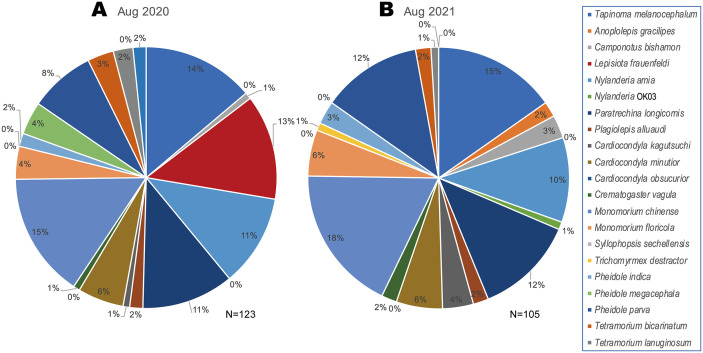
Change in component of the ant community before and after installation of pyriproxyfen. **(A)** August 2020 as the before treatment; **(B)** August 2021 as the after treatment. Each component is shown by the relative frequency of occurrence of each species recorded in 20 surveyed plots by hand collection. No species other than *Lepisiota frauenfeldi* disappeared by the pyriproxyfen effect during the treatment period.

Our attempt to reduce the population of *L*. *frauenfeldi* by selectively inoculating only the target species, while preserving the existing ant community was successful to a certain degree. The results of ant community dynamics observed in this study support our hypotheses for the effectiveness of our target-species-selective control method: (1) it reduced the population density of the target species *L*. *frauenfeldi* below the stable maintenance threshold, (2) minimized impacts on non-target resident ant species within the area, and (3) facilitated the recovery of existing competing ant communities, thereby enhancing biotic resistance contributing to the acceleration of the its eradication and prevention of re-invasion. Out of the 24 non-target ant species collected in the study area, only two species showed moderate decrease in occurrence probability after the pyriproxyfen treatment; however, all declines were smaller than those of the target species *L*. *frauenfeldi*. In fact, the composition of the ant community seems not to change drastically before and after the start of the treatment, except for the disappearance of *L*. *frauenfeldi* (Fig 6). One of the challenges in controlling alien invasive ants is the impact on non-target species [28], and it is assumed that ants sharing the same feeding habits and ecology are particularly susceptible to this impact. At the control site in the early invasion stage studied here, many ant species within the existing community would have competed for food resources with the omnivorous *L*. *frauenfeldi* and for nesting sites with this polygyny, polydomy alien ant. The significant decline in the target species *L*. *frauenfeldi* and the increase in several species within the existing community suggest that the treatment in this study successfully reversed the power balance in competition between the new invasive alien ant and existing species. Gaigher [29] suggested that the impact of baits on non-target species can be reduced by using bait stations that concentrate baits in stations rather than distributing them evenly across the ground. In this study, we took this a step further and placed baits near the nests of the target species *L*. *frauenfeldi*. This strategy takes advantage of the behavior of target species to monopoly food resources and protect them from other ant species and aims to effectively deliver baits to target species while reducing the chance of non-target species taking baits. Despite the specific placement, the two non-target ant species showed a statistically significant reduction in occurrence probability after the treatment (Fig 4). These two species may have won the occupancy competition of the IGR bait over *L*. *frauenfeldi* in some application points and were influenced by Pyriproxyfen. On the other hand, the increases in occurrence probabilities were observed in five species after the pyriproxyfen treatment. These species with increased occurrence may have regained niches from the target ant *L*. *frauenfeldi*, and it may contribute to preventing its re-establishment. The same analyses of survey data up to February 2024 for the 25 ant species show that the increasing trend has also been detected in seven species (Fig 4b). This tendency supports that the niche occupied by *L*. *frauenfeldi* is being filled by other species. The placement of baits to target the nest entrances also drastically reduced the amount of IGR required. Previous pyriproxyfen distribution schemes against ants required about 20 g/ha [30]. In contrast, the total amount of pyriproxyfen used in this study was only 0.86 mg/ha despite bait installation being repeated 35 times in total. The reduction in the amount of chemicals used will directly reduce the environmental impact, which is a side effect of pest control [31].

While this method has advantages in terms of reduced impact on non-target species, maintenance of biological resistance, and reducing the amount of pyriproxyfen use, two challenges were noted in terms of implementation costs. The first is the costs associated with the long-term commitment of experts in the field to search for nest sites and entrances. In the case of *L*. *frauenfeldi*, finding nest entrances was relatively easy because the nesting habitat was an artificial environment, but it may be more difficult in natural habitats. Improved methods for identifying nest locations and dominant foraging areas of target species will be needed as the next step to make this control method more versatile. The second is a time lag of approximately two months for the decline of *L*. *frauenfeldi* after the installation of IGR baits, even though it was earlier than originally expected, then which prolongs the artificial spread prevention measures for the target species. If the target species do not show repellency observed in *L*. *frauenfeldi* in this study, Sakamoto and Goka [26] has suggested that using fipronil agents can shorten the period of control. On the other hand, however, the time lag may be an advantage in helping to allow more widespread penetration of the IGR through inter-nest food-resource sharing.

We need to pay attention that the coordination among local stakeholders, which was emphasized by Wylie [32] and Xu [33], will have a stronger impact on control success under the longer period of spread prevention. A cross-organizational collaboration involving not only research institutions but also several private companies and government agencies contributed to this countermeasure project for *L*. *frauenfeldi* to make it efficient and smooth in this program. The contributions include technical solutions such as a recipe for baits, application methods, effectiveness measurements, also human and financial support, neighborhood monitoring, inter-organizational coordination, legal designation of *L*. *frauenfeldi* and registration on the prefectural invasive species list, and then proper treatments of plant residues and soil left over from the control area, installation of warning signs, and the postponement of a planting project. In addition to intra-national, it was a key contribution to the success of the overall program that the control in the U.S. Army Garrison Okinawa started about eight months after our program, which also successfully reduced *L*. *frauenfeldi* populations in the area. As an issue specific to Okinawa Island, simultaneous control of the invasive species is often not easy in approximately 15% of land area occupied and managed by U.S. military facilities. This eradication program was no exception, and reentry from adjacent restricted areas was a potential cause of control failure, as Wylie et al. [32] pointed out. Therefore, we offered to use our eradication technology at the U.S. Army Garrison Okinawa to make a similar eradication program on the base with the installation of pyriproxyfen bait. This success of the overall control exemplifies the importance of cooperation among organizations existing in the area, as emphasized by Wylie et al. [32] and Xu et al. [33].

## Conclusion

The selective control of generalist target ant species in the early stages of invasion demonstrated in this study provides a new option that promotes communities’ natural biotic resistance. This provides an alternative to the traditional control methods of areal application of chemicals and random bait placement. Given cost considerations, this strategy would be suitable for range-limited implementation, such as in the early stages of invasion or at the distribution expansion edge where target species and existing communities are antagonistic. While customization by species-specific ecology is required, these requirements are relatively modest and could be applied to a wide variety of invasive ant species worldwide. Because this method minimizes impacts on non-target ant species, the trade-off between control and its side effects could be greatly reduced, and it could also be an effective way to control invasive species while protecting rare species.

## Supporting information

S1 Data Raw occurrence records of ant species with survey dates and air temperature. (CSV)

S2 Appendix Inter-nest aggressive test of *Lepisiota frauenfeldi.* (DOCX)

S3 Fig Comparison of the match scores of the aggressiveness test using *Lepisiota frauenfeldi* population in the control area of Naha City. *Paratrechina longicornis* was used for interspecies pairs.
(TIF)
