# Supplementary material for: Maximum effect with minimum impact: A new selective control strategy for the Browsing ant Lepisiota frauenfeldi (Formicidae: Formicinae) minimize the impact on non-target species
Source: PLoS One. 2025 Dec 3;20(12):e0337230. doi: 10.1371/journal.pone.0337230 (PMC12674574; doi:10.1371/journal.pone.0337230)
Supplement: S2 Appendix — (DOCX) [file pone.0337230.s002.docx]

**Supplemental**

**Aggressiveness tests.** In order to obtain the basic ecological information necessary for planning the control of *Lepisiota frauenfeldi* in the study area, exclusivity among nests was investigated by the Aggressiveness tests in June 2020. The entrances of four nests of *Lepisiota frauenfeldi* were located in the study area, each of which was apart more than 30 m, and workers within a radius of 1 m from the entrance were collected as their nestmates. As a comparison, we collected workers of another species *Paratrechina longicornis*, nests sympatrically in the area with similar body size, behavior, and nesting habitat. Two of the workers were entered in a petri dish and observed for five minutes. The aggressiveness of workers in each combination was scored according to the following criteria, and the maximum value was used as the score for the matchup. The scoring criteria were based on modifying Suarez *et al.* (1999). The criteria (points) are the following: ignore (1), touch or antennation (2), evasion as bumping and running away to the opposite direction (3), short attack as biting, pulling, or posing of formic acid spray (4), and longer attack of more than 3 seconds (5). All workers were used only once for the matchup. Scores for each matchup were compared among the three combinations, i.e. intra-nest, inter-nests, and inter-species.

**Result.** A weakness of the exclusivity among the nests of *Lepisiota frauenfeldi* in the study area suggested that this population behaves like a single super colony. The match scores of the aggressiveness test for each combination were 2.15±0.14SE (n=20) for nestmate workers, 2.13±0.11 SE (n=30) for workers of *L*. *frauenfeldi* from the different nests in the study area, and 3.70±0.14 SE (n=20) for between workers of different species, *L*. *frauenfeldi* and *Paratrechina longicornis* (S03 Figure). No significant difference in aggression between the nest-mate workers of *L*. *frauenfeldi* and between the workers of different nests within the area was observed (Steel-Dwass test, p=0.9845). In contrast, aggression between workers of *L*. *frauenfeldi* and *P*. *longicornis* in the same area was significantly higher than those between the nestmates and between the different nests’ workers in the same area (Steel-Dwass test, p<0.0001).

Suarez, A.V., Tsutsui, N.D., Holway, D.A. & Case, T.J. (1999) Behavioral and Genetic Differentiation Between Native and Introduced Populations of the Argentine Ant. *Biological Invasions,* **1,** 43-53.

S03 Figure. Comparison of the match scores of the aggressiveness test using *Lepisiota frauenfeldi* population in the control area of Naha City. *Paratrechina longicornis* was used for interspecies pairs.
