# Supplementary figures and images for: Maximum effect with minimum impact: A new selective control strategy for the Browsing ant Lepisiota frauenfeldi (Formicidae: Formicinae) minimize the impact on non-target species
Source: PLoS One. 2025 Dec 3;20(12):e0337230. doi: 10.1371/journal.pone.0337230 (PMC12674574; doi:10.1371/journal.pone.0337230)

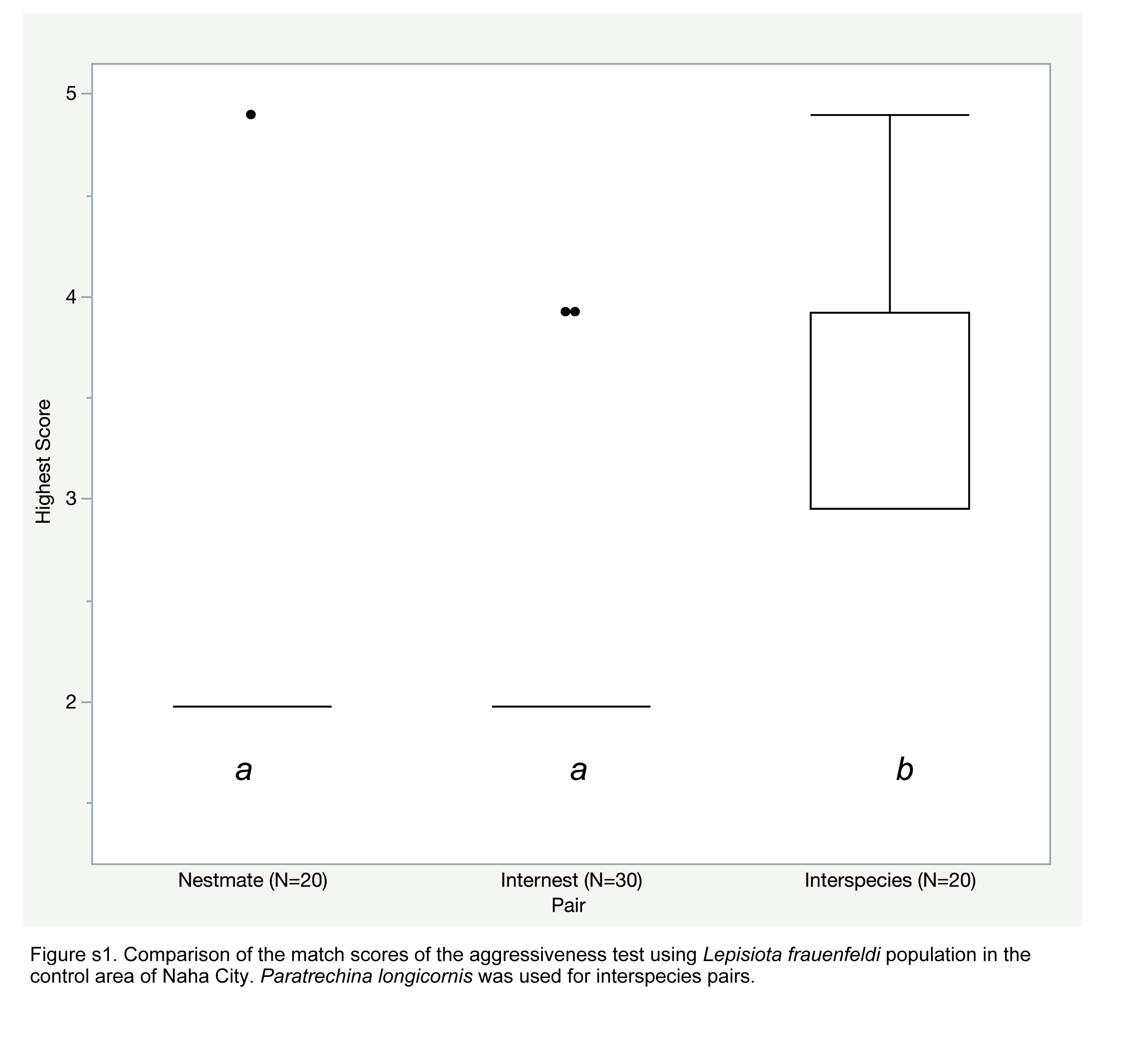

Supplement: S3 Fig — Paratrechina longicornis was used for interspecies pairs. (TIF) [file pone.0337230.s003.tif]
